# Efficacy and Safety of Cerebrolysin as an Adjunct to Mechanical Thrombectomy in Acute Ischemic Stroke: A Systematic Review and Meta‐Analysis of Observational Studies

**DOI:** 10.1002/brb3.71252

**Published:** 2026-03-25

**Authors:** Abdullah Afridi, Fatima Sajjad, Ayesha Arshad, Iqra Shahid, Noor E Fatima, Umama Alam, Rida Khan Hoti, Muhammad Abdullah, Shahbaz Khan, Sajjad GhanimAl‐Badri, Suleman Khan, Muhammad Abu Bakkar, Adam Saeed, Amjad Ali, Laiba Ali Khan, Kamil Ahmad Kamil

**Affiliations:** ^1^ Khyber Medical College Peshawar Pakistan; ^2^ Dow Medical College Karachi Pakistan; ^3^ King Edward Medical University Neela Gumbad Lahore Pakistan; ^4^ Bacha Khan Medical College Mardan Pakistan; ^5^ College of Medicine University of Warith Al‐Anbiyaa Karbala Iraq; ^6^ Department of Medicine Khyber Teaching Hospital Peshawar Pakistan; ^7^ Resident Physician, PGY‐1, Internal Medicine, MBW, HMC Peshawar Pakistan; ^8^ Internal Medicine Department Mirwais Regional Hospital Kandahar Afghanistan

## Abstract

**Background:**

One of the primary causes of morbidity and death is still acute ischemic stroke (AIS). Although mechanical thrombectomy (MT) yields better results, problems such as partial functional recovery persist. By lessening secondary damage and encouraging recovery, the neuroprotective drug, cerebrolysin, may improve the effectiveness of MT. Its safety and efficacy as an adjunct to MT are assessed in this study.

**Methods:**

PubMed, Embase, and Cochrane databases were systematically searched using relevant keywords from inception until June 2025. A total of three studies were included after final screening. Outcomes were reported as symptomatic intracranial hemorrhage, adverse effects, mortality, etc. Interstudy heterogeneity was assessed using *I* ^2^ and *χ* ^2^ statistics (*I* ^2^ > 50% = significant heterogeneity). Statistical calculations were performed using Review Manager 5.4.1 (The Cochrane Collaboration, Copenhagen, Denmark), with a *p*‐value of < 0.05 indicating statistical significance.

**Results:**

By combining data from three studies, this meta‐analysis assessed the safety and effectiveness of cerebrolysin as a supplement to mechanical thrombectomy (MT) in acute stroke. This meta‐analysis pooled data from three observational studies involving a total of 294 patients; 148 were treated with Cerebrolysin in combination with mechanical thrombectomy (Cerebrolysin + MT group), and 146 were treated with mechanical thrombectomy alone (MT group).With a very slight heterogeneity (*I* ^2^ = 2%), Cerebrolysin significantly increased the primary outcome, Good Functional Outcome (mRS 0–3). Among 71 patients in the Cerebrolysin + MT group and 163 in the MT‐alone group, a good functional outcome was achieved in 61 versus 110 patients, respectively (RR: 1.56, 95% CI: 1.25–1.93, *p* < 0.0001). There were sustained protective tendencies and a significant decrease in the incidence of symptomatic intracerebral hemorrhage (sICH) (RR: 0.12, 95% CI: 0.03–0.48, *p* = 0.03). sICH occurred in 4 of 148 patients in the Cerebrolysin group versus 9 of 148 in the control group. Benefits persisted at 1 and 12 months, with mortality being 64% lower in the Cerebrolysin group (RR: 0.36, 95% CI: 0.18–0.68, *p* = 0.02). Mortality was reported in 13 of 246 patients in the Cerebrolysin group versus 41 of 255 in the control group. Across outcomes, there was little heterogeneity. These results indicate cerebrolysin's potential as an adjuvant treatment for stroke management by indicating that it dramatically improves functional recovery, lowers sICH, and decreases mortality post‐MT.

**Conclusion:**

Cerebrolysin shows promise as a useful neuroprotective treatment in stroke care by dramatically improving functional recovery, reducing symptomatic intracranial bleeding, and lowering mortality in acute ischemic stroke patients when used in conjunction with mechanical thrombectomy. Nonetheless, these conclusions are derived from only three observational studies with small sample sizes, which limits the robustness and generalizability of the findings. Further large‐scale randomized trials are warranted to confirm these effects.

## Graphical abstract

● Patients with Ischemic Stroke

● Included 294 Patients

● Cerebrolysin as an Intervention

● Mechanical Thrombectomy as Control

● Good Functional Outcome was significant: 1.56 [1.25, 1.93]

● Symptomatic intracerebral hemorrhage was significant. 0.12 [0.03, 0.48]

● Mortality was significant. 0.36 [0.18, 0.68]

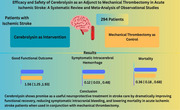

## 1 Introduction

The high rates of morbidity and death associated with acute ischemic stroke (AIS) make it a major global health problem (Donkor 2018). Due to advancements in evidence‐based reperfusion interventions, particularly mechanical thrombectomy (MT), and easy access to neurovascular imaging (angio‐ or perfusion CT [CTA, CTP] or MRI) to inform treatment choices, the standards of care for acute ischemic stroke (AIS) caused by large vessel occlusion (LVO) have changed dramatically in recent years (Turc et al. 2023). A significant improvement in the management of AIS was seen in 2015 as a result of the release of innovative randomized controlled trials (RCTs) that contrasted endovascular and medical therapies. These investigations showed that mechanical thrombectomy (MT) for large vascular occlusion (LVO) can effectively lower mortality and enhance results (Berkhemer et al. 2015; Campbell et al. 2015; Saver et al. 2015). The most effective treatment for acute ischemic stroke is recanalization therapy. For the past five years, intravenous thrombolysis with recombinant tissue plasminogen activator (rtPA) has been the first‐choice treatment for major artery occlusions of the anterior circulation, alongside thrombectomy, since it was approved (Powers et al. 2019). Effective recanalization and the restoration of pre‐stroke functional capacities still differ significantly, despite the progress made in MT. The mortality rate of 15.3% is still concerning. Additionally, only 46 percent of patients with anterior circulation AIS who had endovascular treatment achieved functional independence within 3 months (Goyal et al. 2016). Given the above uncertainties, it is essential to focus not only on improvements in vascular recanalization but also on cerebroprotection, which refers to methods to preserve neurological function and minimize infarct growth by protecting viable brain tissue during the acute phase of stroke. To improve the prognosis of stroke patients, it will be essential to investigate quick and efficient cerebroprotection techniques to supplement MT (Lyden 2021; Paul and Candelario‐Jalil 2021). Research has demonstrated that Cerebrolysin, a multimodal neuroprotective drug, improves the safety and effectiveness of mechanical thrombectomy (MT) in both the acute ischemic stroke (AIS) and recovery stroke phases in a subset of patients with good collateral status, as well as effective recanalization (Staszewski et al. 2022). After ischemia damage, cerebrolysin prevents apoptosis, increases neuronal survival, fosters brain plasticity and recovery, and lessens excitotoxic cascades by lowering calcium excess and reperfusion (Heiss et al. 2012). Its anti‐inflammatory and antioxidant qualities also help to protect the delicate penumbral tissue and lessen secondary damage (Zhang et al. 2019). By decreasing inflammatory proteins and increasing tight junction proteins, cerebrolysin preserves the blood‐brain barrier and improves the permeability of fibrin‐impaired cerebral endothelial cells, hence lowering hemorrhagic transformation, according to a recent in vitro study (Teng et al. 2021).

This study aims to evaluate the safety and effectiveness of cerebrolysin as a mechanical thrombectomy adjuvant in enhancing functional outcomes and lowering mortality in ischemic stroke.

## 2 Methods

### 2.1 Study Design and Protocol Registration

This systematic review adhered to the guidelines set by the Cochrane Collaboration (Cochrane Handbook for Systematic Reviews of Interventions | Cochrane Training 2025) and the Preferred Reporting Items for Systematic Reviews and Meta‐Analysis (PRISMA) framework (Page et al. 2021). It encompassed the study design, stepwise implementation, analysis, and presentation of findings. Additionally, the study protocol was registered in the International Prospective Register of Systematic Reviews (PROSPERO) under registration number CRD420251067994.

### 2.2 Search Strategy and Databases

An electronic search of PubMed, Embase, and Cochrane was conducted, covering all available entries from their inception to June 2025, without any language restrictions. The following keywords were used: “acute ischemic stroke,” “cerebrolysin and mechanical thrombectomy,” and “mechanical thrombectomy.” A detailed search strategy for each database is given in Supplementary Table S2.

### 2.3 Study Selection and Eligibility Criteria

All studies identified through the electronic search were imported into Rayyan software for screening, and duplicates were eliminated. The remaining studies were initially assessed based on their titles and abstracts. Full‐text articles were selected for further evaluation if either reviewer considered the abstract potentially relevant. Two independent reviewers (A. A. and F. S.) assessed the eligibility of each study based on predefined inclusion criteria. Any disagreements were resolved through discussion and consultation with a third reviewer (U. A.).

Studies were included if they met the following criteria: (1) involved patients with acute ischemic stroke, (2) cerebrolysin and mechanical thrombectomy as intervention, (3) mechanical thrombectomy alone as control, (4) observational studies, and (5) reported at least one relevant outcome.

Exclusion criteria included (1) overlapping populations, defined by shared institutions and recruitment periods; (2) populations outside the scope of interest; (3) republished literature; (4) protocols without reported results; (5) reviews, abstracts, case reports, case series, background articles, expert opinions, or in vivo/in vitro studies; (6) repeated data from the same clinical trial; or (7) absence of a control group.

Although some relevant randomized controlled trials (RCTs) investigating Cerebrolysin in stroke exist, they were excluded from this meta‐analysis due to differences in intervention protocols and outcome measures that were not compatible with the context of the topic. Only observational studies directly comparing Cerebrolysin and MT versus MT alone were included to ensure methodological consistency and relevance to the research question.

### 2.4 Data Extraction and Outcomes

Two authors (A. A. and U. A.) extracted data from the included studies into an Excel sheet using a pre‐piloted form. Baseline data included age, sex, hypertension, diabetes, hyperlipidemia, smoking, Thrombolysis in Cerebral Infarction score, and occlusion site at the middle cerebral artery. The primary outcomes of this study included good functional outcomes (mRS score 0–3) and symptomatic intracranial hemorrhage, and the secondary outcome included mortality. See Supplementary Table S1 for a detailed definition of outcomes.

### 2.5 Quality Assessment

For quality assessment, the Risk of Bias in Non‐Randomized Studies of Interventions (ROBINS‐I) tool (Schünemann et al. 2019) was applied, assessing bias across seven domains: confounding, selection of participants, classification of interventions, deviations from intended interventions, missing data, measurement of outcomes, and selection of reported results. The overall risk of bias was classified as low, moderate, severe, or critical.

Two reviewers (A. A. and U. A.) independently assessed the risk of bias, resolving disagreements through discussion, with a third reviewer (F. S.) consulted if necessary. This systematic assessment ensured the reliability and validity of the included studies.

### 2.6 Statistical Analysis and Sensitivity Analysis

Review Manager 5.4 was used to perform statistical analysis. Treatment effects for binary outcomes were compared using a pooled risk ratio (RR) with 95% confidence intervals (CI), while continuous outcomes were analyzed using mean differences (MD) with 95% CI. The Cochran *Q* test and *I* ^2^ statistics were used to assess heterogeneity, with *P*‐values < 0.10 and *I* ^2^ > 50% considered indicative of significant heterogeneity (Higgins et al. 2003). The DerSimonian and Laird random‐effects model was applied to all outcomes (DerSimonian and Laird 1986). A *p*‐value of < 0.05 indicates statistical significance for clinical endpoints. The stability of the pooled estimates was assessed through a leave‐one‐out analysis, where each study was sequentially removed, and the remaining dataset was re‐analyzed to ensure that no single study unduly influenced the aggregated effect sizes. Moreover, due to the limited number of included studies (*n* = 3), formal assessment of publication bias (e.g., funnel plot analysis) was not performed, as such methods are considered unreliable with small sample sizes.

## 3 Results

### 3.1 Searched Results

**FIGURE 1:**
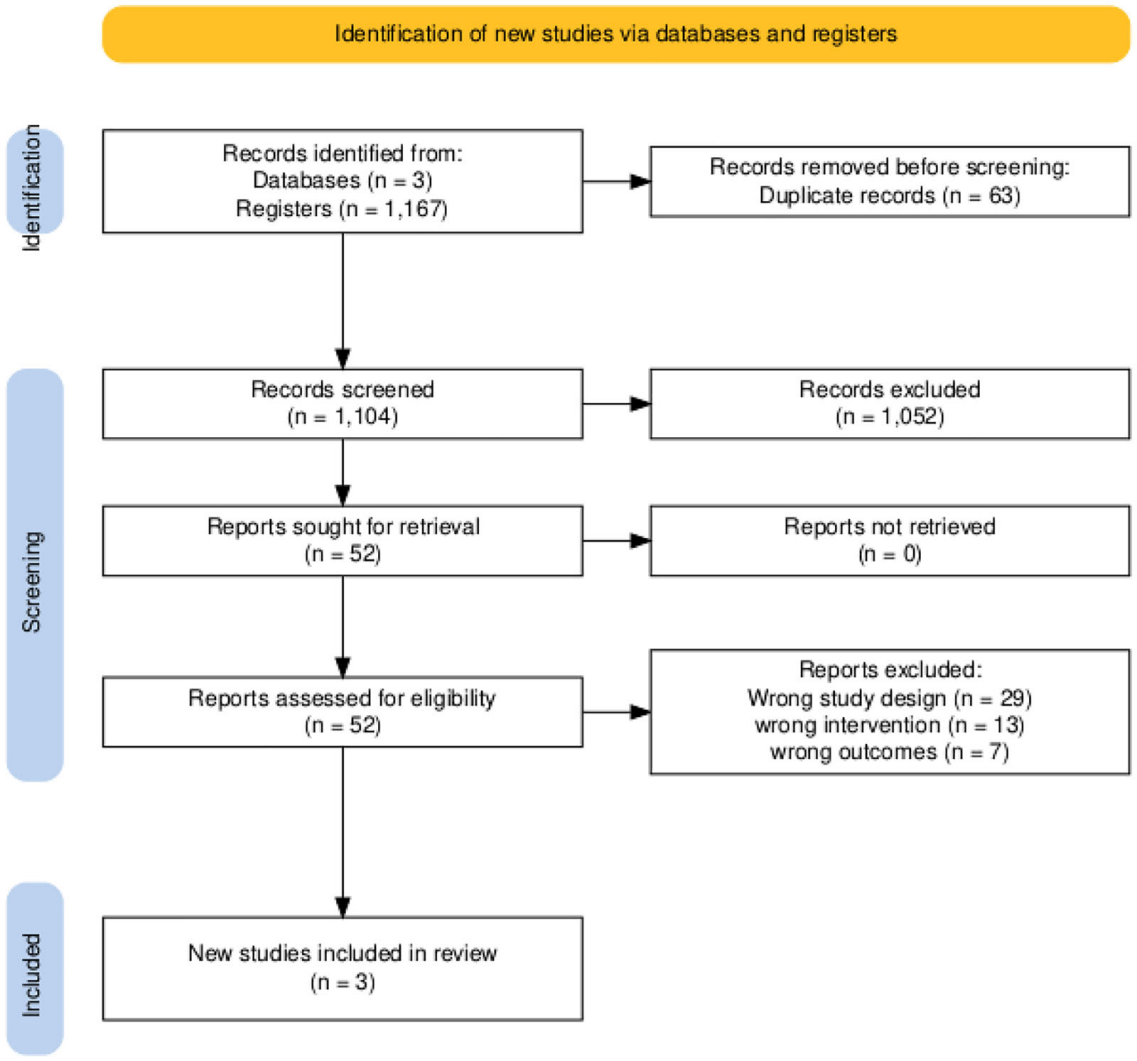
PRISMA flowchart.

A total of 1167 records were identified through database and register searches: 962 from PubMed, 125 from Cochrane, and 80 from Embase using a search string including all the relevant MeSH terms. After the removal of duplicates, 1104 articles remained for title and abstract screening. Following the screening process, 1052 articles were excluded, and 52 were assessed for eligibility. Three articles were included in our meta‐analysis (Staszewski et al. 2022; ElBassiouny et al. 2025; Poljakovic et al. 2021). Details of the screening process are given in Figure 1.

### 3.2 Study Characteristics

Our meta‐analysis included three observational studies involving a total of 294 patients, with 148 in the Cerebrolysin + MT group and 146 in the MT group. The average age of participants was 69 years in the Cerebrolysin and MT group and 68 years in the MT group. The proportion of male participants was 73 in the Cerebrolysin and MT group and 84 in the MT group. Hypertension was present in 69% of patients in the Cerebrolysin + MT group, compared to 65% in the MT group. The prevalence of diabetes was 31% in the Cerebrolysin + MT group and 39% in the MT group. Hyperlipidemia was observed in 41% of patients in the Cerebrolysin + MT group and 30% of those in the MT group. The proportion of smokers was 38% in the Cerebrolysin + MT group and 35% in the MT group. The studies were conducted in Egypt, Croatia, and Poland. Detailed baseline characteristics are mentioned in Tables 1 and 2.

**TABLE 1:** Study characteristics.

Author, Year	Recruitment period	Country	Type of Study	Intervention	Control	Interventional Dose and route of administration	Sample size		Inclusion criteria
							Cerebrolysin + MT	MT	
ElBassiouny, 2025	Oct 2021‐ Jan 2024	Egypt	Cohort	Cerebrolysin infusion after MT	Mechanical Thrombectomy	30 mL of Cerebrolysin diluted in 100 mL of 0.9% normal saline was administered intravenously	75	75	‐Cardioembolic AIS with LVO ‐ Successful MT with mTICI 2b or 3 ‐ Age 18–80 ‐ NIHSS ≥10
Poljakovic, 2021	Jan 2018‐ Jan 2019	Croatia	Cohort	Cerebrolysin after recanalization	Mechanical Thrombectomy	30 mL of Cerebrolysin administered intravenously	23	21	‐Acute ischemic stroke ‐ NIHSS ≥8 ‐ Indication for thrombolysis and/or thrombectomy ‐ Futile early recanalization (clinical or imaging‐based)
Staszewski, 2025	Jun 2021‐ Dec 2023	Poland	Cohort	Cerebrolysin after mechanical thrombectomy (MT) treatment	Mechanical Thrombectomy	30 mL of Cerebrolysin mixed with 250 mL of saline was administered intravenously	50	50	‐AIS with LVO in the anterior circulation ‐ mTICI ≥2b post‐MT ‐ CTA collateral score 2–3 ‐ NIHSS ≥5 post‐MT ‐ Able to receive Cerebrolysin within 8 h of stroke onset

**Abbreviations**: AIS, acute ischemic stroke; CTA, CT Angiography; LVO, large vessel occlusion; MT, mechanical thrombectomy; mTICI, modified Thrombolysis In Cerebral Infarction score; NIHSS, National Institutes of Health Stroke Scale.

**TABLE 2:** Patient characteristics.

	ElBassiouny, 2025		Poljakovic, 2021		Staszewski, 2025	
	MT + Cerebrolysin	MT	MT + Cerebrolysin	MT	MT + Cerebrolysin	MT
Age (yrs) Mean (SD)	59.2 (15.4)	61.7 (11.4)	76 (9.8)	72 (10.6)	72 (18)	71 (17)
Male *N* (%)	31 (41)	45 (60)	14 (60.8)	10 (47.6)	28 (56)	29 (58)
Hypertension *N* (%)	45 (60)	38 (50)	NR	NR	41 (82)	43 (86)
Diabetes *N* (%)	27 (36)	36 (48)	NR	NR	12 (24)	13 (26)
Hyperlipidemia *N* (%)	24 (32)	14 (18.7)	NR	NR	27 (54)	24 (48)
Smoking *N* (%)	26 (34.7)	25 (33.3)	NR	NR	22 (44)	19 (48)
TICI 3 *N* (%)	NR	NR	6 (26)	3 (14)	28 (56)	21 (43)
MCA (M1 segment) Occlusion site	46 (65.7)	53 (70.7)	NR	NR	40 (80)	38 (76)

**Abbreviations**: MCA, Middle cerebral artery; MT, mechanical thrombectomy; SD, Standard Deviation; TICI, Thrombolysis In Cerebral Infarction score.

### 3.3 Risk of Bias

The included studies were assessed using the ROBINS‐1 tool. Overall, the studies showed low to moderate risk of bias. The domain‐level evaluation revealed that all three studies demonstrated low risk of bias across most domains, particularly in the classification of interventions, missing data, and measurement of outcomes. First, Staszewski^2022^ (2025) showed low risk of bias across all seven domains, including confounding, participant selection, classification of interventions, deviations from intended interventions, missing data, outcome measurement, and selection of reported results. This study was judged to have an overall low risk of bias. Second, Poljakovic^2021^ (2025) exhibited a moderate risk of bias due to confounding and a moderate risk in the selection of reported results, while all other domains were rated as low risk. The overall judgment was moderate risk of bias. Lastly, ElBassiouny^2025^ (2025) also showed moderate risk due to confounding, with low risk across the remaining domains. The overall risk of bias was considered moderate. A detailed assessment of the risk of bias is presented in Supplementary Figures S1 and S2.

### 3.4 Outcomes

#### 3.4.1 Primary Outcomes

##### 3.4.1.1 Good Functional Outcome

**FIGURE 2:**
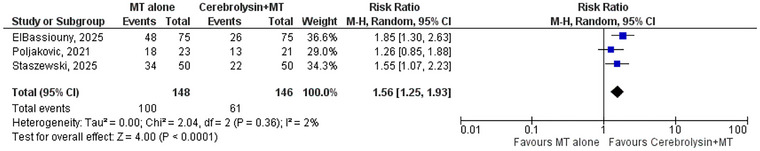
Forest plot of good functional outcome.

A good functional outcome, assessed by the modified Rankin Scale (mRS) score of 0 to 3, was reported in all three studies, consisting of 148 patients in the intervention group and 146 patients in the control group. The pooled results showed a significant improvement in outcomes, yielding a combined Risk Ratio (RR) of 1.56 [95% CL: 1.25–1.93, *p* < 0.0001]. This indicates that patients receiving Cerebrolysin alongside MT were 56% more likely to achieve good functional outcomes compared to those treated with MT alone. Individually, the study by ElBassiouny et al. (2025) reported an OR of 1.85 [95% CL: 1.30–2.63], followed by the study by Staszewski et al. (2022) with an RR of 1.55 [95% CL: 1.07–2.23], while Poljakovic et al. (2021) reported a non‐significant RR of 1.26 [95% CL: 0.85–1.88]. More importantly, there was a lack of statistical heterogeneity among the studies (*χ* ^2^ = 2.04, df = 2, *p* = 0.36; *I* ^2^ = 2%), indicating minor variability in findings across the included trials. These findings support the potential efficacy of Cerebrolysin as an adjunct to MT in improving post‐stroke functional recovery (Figure 2).

##### 3.4.1.2 Symptomatic Intracerebral Hemorrhage (sICH)

**FIGURE 3:**
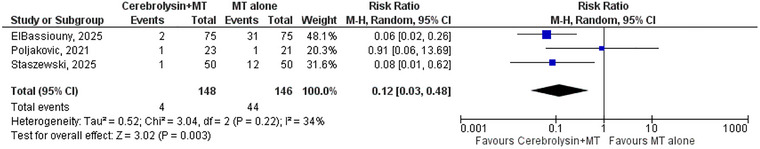
Forest plot of symptomatic intracerebral hemorrhage (sICH).

The studies also report data on symptomatic intracerebral hemorrhage. A total of 44 sICH events occurred in the control group, compared to just four in the intervention group. The pooled risk ratio (RR) for sICH was 0.12 [95% CL = 0.03, 0.48], showing an 88% reduction in risk of sICH with Cerebrolysin use, which was statistically significant (*p* = 0.03). While there was moderate heterogeneity (*I* ^2^ = 34), it did not reach statistical significance (*p* = 0.22), and the direction of effect favored the intervention group in two of the three studies. Studies (ElBassiouny et al. 2025; Staszewski et al. 2022) showed robust protective effects (RR: 0.06 and 0.08, respectively), while Poljakovic et al. (2021) demonstrated a neutral result with a wide confidence interval (RR: 0.91; 95% CL: 0.06, 13.69) (Figure 3).

#### 3.4.2 Secondary Outcome

##### 3.4.2.1 Mortality

**FIGURE 4:**
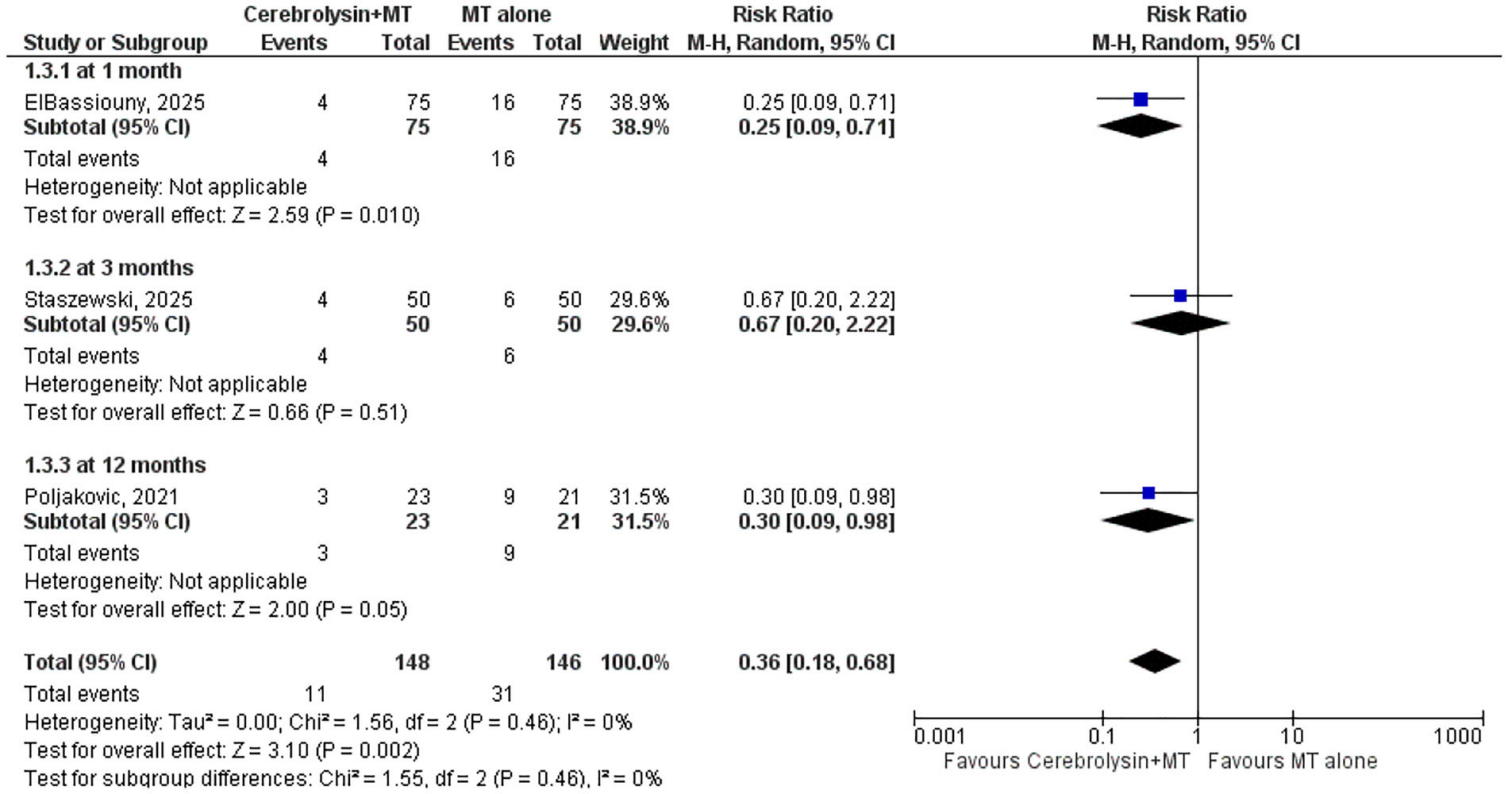
Forest plot of mortality.

Pooled analysis from three studies revealed a significant reduction in mortality associated with the use of Cerebrolysin. The overall RR was 0.36 [95% CL: 0.18, 0.68], indicating a 64% relative reduction in mortality in the intervention group compared to the control group. The effect was statistically significant (p = 0.02), and no heterogeneity was observed across studies (*I^2^* = 0%, *p* = 0.46), suggesting consistent findings. Subgroup analyses were conducted to explore mortality outcomes across different follow‐up durations, at approximately 1 month, 3 months, and 12 months. Significant reductions in mortality were observed at 1 month (RR: 0.25; 95% CI: 0.09–0.71; *p* = 0.01) and 12 months (RR: 0.30; 95% CI: 0.09–0.98; *p* = 0.05). No significant difference was found at 3 months (RR: 0.67; 95% CI: 0.20–2.22; *p* = 0.51), likely due to limited sample size and zero‐event data in both groups. The test for subgroup differences was non‐significant (*p* = 0.46), indicating no statistical interaction between follow‐up duration and treatment effect. This suggests a generally consistent mortality benefit across time points, although the strength of evidence varies. These time points were selected based on the availability of reported data and reflect early, intermediate, and long‐term follow‐up intervals commonly used in stroke outcome studies (Figure 4).

### 3.5 Overall Interpretation

The results of this meta‐analysis suggest that Cerebrolysin, when added to standard medical therapy, is associated with a notable reduction in both mortality and incidence of sICH, showing its potential role in improving survival and safety outcomes in acute neurological care. Moreover, the observed improvements in functional outcomes further support its role as an adjunctive neuroprotective therapy, suggesting that the patient's survival may be accompanied by better long‐term functional recovery. However, while the pooled analysis demonstrates statistically significant reductions in mortality and sICH, the relatively small sample size (*n* = 294) across the included studies may limit the precision of these estimates. Even though confidence intervals are significant, they should be interpreted with caution, as smaller datasets can exaggerate effect sizes and reduce generalizability. Further large‐scale, randomized trials are needed to validate these findings.

## 4 Discussion

The findings of this meta‐analysis highlight Cerebrolysin as a neuroprotective adjunct in the management of AIS treated with MT. The statistically significant reductions in sICH and mortality, along with notable improvements in functional outcomes, highlight Cerebrolysin's potential benefit and raise important questions about the broader clinical implications of adjunctive neuroprotective therapy.

Although MT is effective in restoring perfusion, it can lead to a catastrophic complication: intracerebral hemorrhage (Jain 2019). Studies have shown that approximately 40% of patients develop intracerebral hemorrhage post‐thrombectomy (Bracard et al. 2016; Lee et al. 2023; Blanc et al. 2020). The reduction in sICH observed with Cerebrolysin's use aligns with its well‐documented preclinical effects in blood barrier stabilization and attenuation of reperfusion injury (Zhang et al. 2010; Hartwig et al. 2014; Sarode et al. 2023; Bornstein et al. 2018; Strilciuc et al. 2021). Moreover, tissue damage due to oxidative stress and inflammation in patients undergoing MT is probably lessened by cerebrolysin's antioxidative and anti‐inflammatory qualities, which align with its well‐established safety benefits (Zhang et al. 2019). This is important since sICH can counteract the advantage of recanalization and is still an important limiting factor in AIS treatment outcomes.

Similarly, the decrease in mortality maintained during the early and late follow‐up periods may not only be due to Cerebrolysin's neuroprotective benefits but also due to its systemic effects, such as reduced secondary complications (e.g., edema (Yang et al. 2016), hemorrhagic transformation, or infection). An in vitro study published in 2021 showed that Cerebrolysin protects the blood‐brain barrier by reducing inflammation and increasing tight junction proteins, thereby reducing hemorrhagic transformation (Teng et al. 2021). According to these results, Cerebrolysin may provide several advantages by stabilizing patients during the vulnerable post‐reperfusion phase. This interpretation is supported by the absence of heterogeneity in mortality outcomes across studies, indicating a potential consistent biological effect.

Moreover, the improvement in functional outcomes (mRS 0–3) in the intervention group may be attributed to Cerebrolysin's neuroprotective and neurorestorative properties. Cerebrolysin is known to mimic the actions of endogenous neurotrophic factors, promoting neuronal survival and synaptic remodelling (Baskys and Wojtowicz 1994; Juárez et al. 2011; Alcántara‐González et al. 2012), reducing excitotoxicity (Sarode et al. 2023), and supporting neuroplasticity, all of which are crucial in the acute and subacute phases of ischemic stroke recovery (Majithia 2024; Grefkes and Fink 2020; Chanubol and Lertbutsayanukul 2021). A previous study reveals that patients who were more severely affected at baseline tended to respond better to cerbrolysin, and had less severe stroke deficits or greater improvements in motor functions (Muresanu et al. 2016; Vilenskiĭ et al. 2000). These effects may help preserve brain tissue that is infarcted but not yet ischemic. Moreover, when combined with MT, which restores blood flow and inhibits or slows down the ischemic cascade, thereby limiting further damage (Neurosurgeons of New Jersey—Cerebrovascular Team 2022; Weller et al. 2022), Cerebrolysin, on the other hand, enhances recovery and regeneration. The consistency of the results across studies, as indicated by minimal significant statistical heterogeneity, supports a potentially reliable biological mechanism rather than chance. The lack of statistical significance in the Poljakovic (2021) study is likely due to its small sample size and limited power, rather than a true absence of effect, as the direction of benefit was still consistent. Furthermore, defining a good functional outcome as mRS 0–3 emphasizes not just survival but meaningful recovery, including independence in daily activities and mobility (Zihni et al. 2022; Saver et al. 2021), which is particularly important in assessing real‐world clinical value. These findings suggest that Cerebrolysin, when administered in conjunction with MT, could play a significant role in enhancing recovery by not only preventing further neuronal injury but also facilitating post‐stroke neurorehabilitation processes.

In the past, many neuroprotective drugs that showed promising results in laboratory studies failed to show benefits in clinical settings. This was often due to issues such as patient selection, timings, or dose of the treatment, or inaccurate combination with reperfusion therapies, such as thrombolysis (Fiani et al. 2021; Beghi et al. 2021). However, this situation is much easier to handle with the use of MT. MT has made it easier to open blocked vessels, and modern imaging techniques help doctors better identify areas of the brain that can be saved. These improvements offer a better chance for neuroprotective drugs like Cerebrolysin to make a real difference, especially when used in carefully selected patients at the right stage of treatment.

Nonetheless, these results must be considered alongside prior evidence. The Cochrane review by Ziganshina et al. found insufficient support from randomized trials for the routine administration of Cerebrolysin in acute ischemic stroke, indicating negligible benefits on mortality or major complications and a potential rise in non‐fatal serious adverse outcomes (Ziganshina et al. 2023). Moreover, despite earlier encouraging findings, the large CASTA trial failed to demonstrate any significant benefit of Cerebrolysin over placebo as well Heiss (2012). Another study, the CARS trial, suggested modest functional gains with Cerebrolysin during early rehabilitation. However, its exploratory nature, small sample size, and disproportionately poor placebo outcomes limit the strength and generalizability of the reported benefits (Muresanu et al. 2016).

This meta‐analysis also illustrates the limitations of existing evidence. While all three studies were generally low in bias, their small sample sizes, varying protocols, and inconsistent reporting of secondary outcomes limit the precision and generalization of our findings. Furthermore, the inclusion of only three observational studies considerably restricts the overall robustness and external validity of our conclusions, as observational studies are inherently more prone to confounding and bias than randomized controlled trials (RCTs), which represent the highest standard of clinical evidence. The absence of RCTs specifically evaluating Cerebrolysin in the context of mechanical thrombectomy may affect the strength and reliability of our conclusions. Additionally, publication bias could not be formally assessed due to the small number of studies, which may limit the comprehensiveness of our findings. Lastly, the heterogeneous definitions of functional outcomes and the absence of detailed subgroup analysis make it difficult to understand which patients benefit the most.

While this meta‐analysis supports the safety and mortality benefits of Cerebrolysin in AIS patients undergoing MT, it also underscores the need for a more complex understanding of neuroprotective agents and their effects on these outcomes. These findings advocate for a reexamination of how neuroprotective strategies are integrated into acute stroke care. However, given that the current evidence is based solely on three non‐randomized, observational studies with heterogenous results and geographically limited populations, these conclusions should be interpreted with caution. Future research should focus on finding markers, such as stroke size and blood flow patterns, that can help guide when and for whom to use Cerebrolysin. Studies should also look at long‐term recovery, quality of life, and treatment costs to better understand its overall impact. Giving Cerebrolysin early and alongside thrombectomy may also improve its effectiveness, but large‐scale randomized controlled trials are needed before it can be recommended for routine stroke care.

## Author Contributions

All authors have made substantial intellectual contributions to this work and approved the final manuscript. Individual contributions are specified below: **Conceptualization**: Abdullah Afridi, Fatima Sajjad, and Kamil Ahmad Kamil. **Methodology**: Abdullah Afridi, Fatima Sajjad, and Umama Alam. **Data Curation**: Abdullah Afridi, Umama Alam, and Ayesha Arshad. **Formal Analysis**: Abdullah Afridi and Umama Alam. **Investigation**: Abdullah Afridi, Fatima Sajjad, and Iqra Shahid. **Writing – Original Draft Preparation**: Abdullah Afridi, Fatima Sajjad, and Noor E. Fatima. **Writing – Review and Editing**: All authors. **Visualization (Figures/Tables)**: Umama Alam and Rida Khan Hoti. **Supervision**: Kamil Ahmad Kamil and Muhammad Abu Bakkar. **Project Administration**: Kamil Ahmad Kamil (Corresponding Author).

## Funding

The authors have nothing to report.

## Conflicts of Interest

The authors declare no conflicts of interest.

## Supporting information




**Supporting Materials**: brb371252‐sup‐0001‐SuppMat.docx

## Data Availability

The data that supports the findings of this study are available in the supplementary material of this article.
