# Supplementary material for: Efficacy and Safety of Cerebrolysin as an Adjunct to Mechanical Thrombectomy in Acute Ischemic Stroke: A Systematic Review and Meta‐Analysis of Observational Studies
Source: Brain Behav. 2026 Mar 25;16(3):e71252. doi: 10.1002/brb3.71252 (PMC13045362; doi:10.1002/brb3.71252)
Supplement: Supplementary file 1 — Supporting Materials: brb371252‐sup‐0001‐SuppMat.docx [file BRB3-16-e71252-s001.docx]

| **Primary Outcomes** | **Outcome** | **Definition** |
| --- | --- | --- |
| 1 | Good Functional Outcome | A favorable clinical result typically defined by a **modified Rankin Scale (mRS) score of 0-3** (indicating no or mild disability), representing a significant improvement in daily function after treatment. |
| 2 | Symptomatic Intracerebral Hemorrhage (sICH) | Intracerebral bleeding accompanied by neurological deterioration, typically confirmed by imaging and clinical assessment.. |
| **Secondary Outcomes** | **Outcome** | **Definitions** |
| 1 | Mortality | All-cause death occurring within the follow-up period, commonly reported at 90 days post-treatment. |

**Supplementary Table S1:** Definition of Outcomes

| Database | Search Date | Search Strategy / Query |
| --- | --- | --- |
| PubMed | June 2025 | ("acute ischemic stroke"[MeSH Terms] OR "acute ischemic stroke" OR "ischemic stroke" OR "cerebral infarction") AND ("Cerebrolysin"[MeSH Terms] OR "Cerebrolysin") AND ("mechanical thrombectomy"[MeSH Terms] OR "endovascular thrombectomy" OR "thrombectomy" OR "endovascular treatment") |
| Embase | June 2025 | ('acute ischemic stroke'/exp OR 'ischemic stroke' OR 'cerebral infarction') AND ('cerebrolysin'/exp OR 'cerebrolysin') AND ('mechanical thrombectomy'/exp OR 'endovascular thrombectomy' OR 'endovascular treatment' OR 'thrombectomy') |
| Cochrane | June 2025 | ("acute ischemic stroke" OR "ischemic stroke" OR "cerebral infarction") AND ("Cerebrolysin") AND ("mechanical thrombectomy" OR "endovascular thrombectomy" OR "endovascular treatment") |

**Supplementary Table S2:** Search Strategy for Each Database


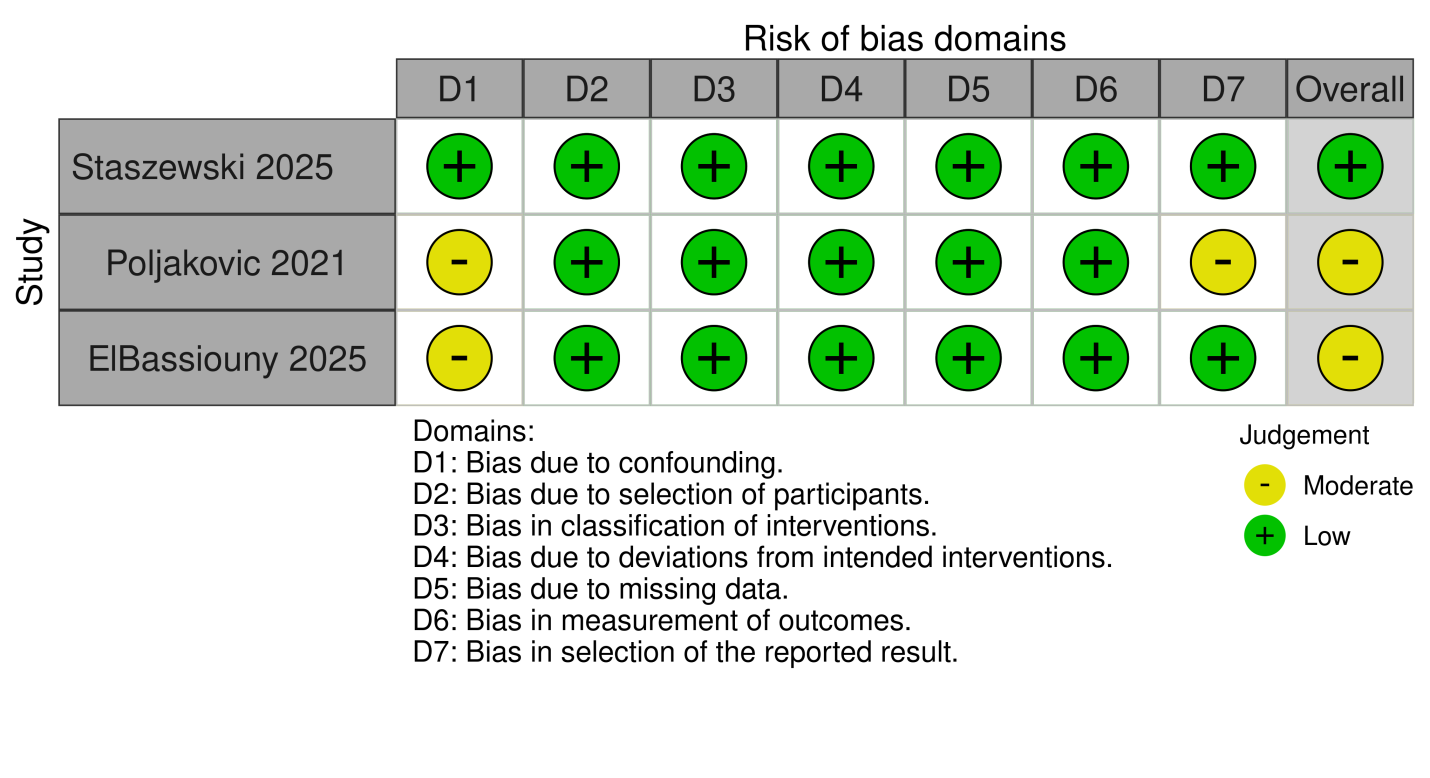


**Supplementary Figure S1:** Traffic plot of Risk of Bias Assessment using ROBINS-1 tool


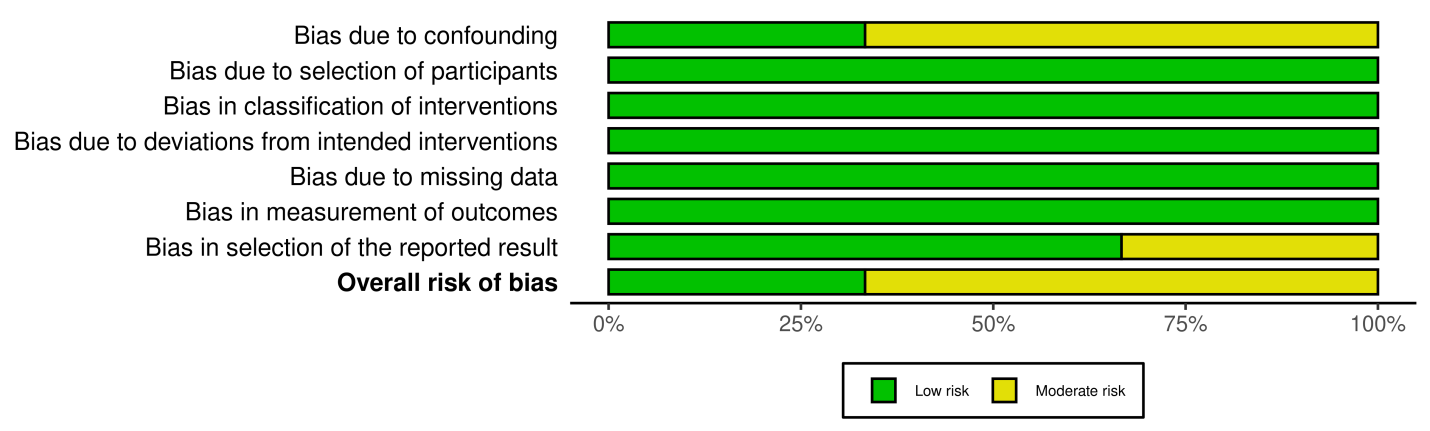


**Supplementary Figure S2:** Summary Plot of Risk of Bias Assessment
